# Eating Habits and Preferences of Polish Women Undergoing Treatment for Breast Cancer

**DOI:** 10.31557/APJCP.2019.20.6.1803

**Published:** 2019

**Authors:** Anna Demuth, Urszula Czerniaka

**Affiliations:** *Department of Anthropology and Biometry, University School of Physical Education in Poznań, Królowej Jadwigi str. 27/39, 61-871 Poznań, Poland. *

**Keywords:** Eating habits, women after mastectomy, prevention

## Abstract

**Objective:**

The objective of the study is the assessment of the nutrition as well as the eating habits and preferences of Polish women undergoing breast cancer treatment.

**Material and methods:**

The study included women undergoing breast cancer treatment aged over 50 years. Data were collected on 112 respondents, residents of a large city, for whom the average time elapsed since the completion of treatment was 5 years. Their eating habits and preferences were determined on the basis of the frequency of consumption of products with potentially beneficial and adverse effects on health, which made it possible to determine the pro-Healthy Diet Index-10 (pHDI-10) and non-Healthy Diet Index-14 (nHDI-14). The intensity of properties beneficially and adversely affecting health was assessed in three intervals: small, moderate, and large. On the basis of the BMI index persons with normal body proportions were identified as well as those above the norm. The effect of lifestyle and socioeconomic status on pHDI-10 and nHDI-14 was determined.

**Results:**

More than half of the studied women had normal body weight and 39% were overweight. One in three women was characterised by a low level of physical activity. The participants of the study followed diets with a low intensity of unhealthy properties with a weak influence of protective properties of nutrition. A significant, adverse effect of the nutritional knowledge and health self-assessment on pro-Healthy Diet Index-10 and non-Healthy Diet Index-14 was demonstrated. Bad habits related to lifestyle (excessive energy consumption/overweight, smoking cigarettes in the past) contributed to overconsumption of unhealthy products/dishes.

**Conclusion:**

The demonstrated dietary mistakes indicate that actions aiming to promote benefits of undertaking behaviour beneficial for health should also be carried out among women with breast cancer diagnosis after the completion of treatment.

## Introduction

The World Cancer Research Fund and the American Institute for Cancer Research (2007) emphasises that the incidence of malignant cancers increases with the social and economic development. In Poland the dynamics of increase in incidence and deaths caused by breast cancers is one of the highest in Europe. This is due to many limitations in screening tests and an insufficient oncology awareness among patients. Therefore, in Poland breast cancer ranks first in incidence of cancers in women. Most incidents of breast cancer (80%) occur after 50 years of age, whereas half of the cases are diagnosed between the age of 50 and 69 (Didkowska et al., 2017).

Results of research indicate that both the level of women’s knowledge about breast cancer prevention and pro-health attitudes in this respect are insufficient in Poland (Bogusz et al., 2016). As breast cancer risk factors women most often list non-modifiable factors, e.g. genetic factors (Zuzak et al., 2018). A significant percentage of the respondents do not know that there are risk factors which every women can modify, such as an inappropriate diet, excessive body mass, excessive alcohol consumption. Also physical exercise is said to have an unquestionable effect on the possibility and rate of development of breast cancer (Virdine et al., 2013; Thomson et al., 2014; Dieli-Conwright et al., 2016). The situation necessitates intensification of prevention activities in order to decelerate and decrease the speed of incidence of breast cancer by modification or elimination of adverse risk factors.

On the basis of many years of observation and research a large number of factors increasing breast cancer risk were identified. The data published by the International Agency for Research on Cancer (IARC) indicates that one quarter of breast cancer cases are linked to inappropriate diet, whereas pro-healthy lifestyle may contribute to lowering the risk of the disease by as much as 50%.

The nutritional factors which modify the risk of breast cancer development are: consumption of monosaccharides, the amount and composition of fatty acids in the diet, consumption of fruit and vegetables, and the energetic value of the diet.

Therefore, it seems that propagation of a healthy diet and spreading information on the prevention of cancers may significantly contribute to the lowering of their incidence. In Poland, in spite of many publications describing cancer risk factors, there are few analyses and studies available the results of which are based on standardised research tools and thus present the phenomenon in question. It seems that getting to know breast cancer risk factors may significantly increase women’s chances of avoiding the disease and, if the disease does occur, increase their chances of overcoming the disease, through appropriate modifications (Dieli-Conwright et al., 2016). It is therefore particularly important to develop appropriate attitudes which will make it possible to undertake all actions for the benefit of one’s own health and the health of others. A healthy person is a determinant of a country’s social and economic development.

*Objective of the study*

The objective of the study was the assessment of nutrition and eating habits and preferences of Polish women undergoing treatment for breast cancer.

## Materials and Methods

The study included 112 women undergoing breast cancer treatment (Amazons), gathered in the Poznań Amazons Association. The studied women had undergone radical surgery and/or chemotherapy, radiotherapy, hormonal therapy. An average time that elapsed between the completion of the treatment and the study exceeded five years. The respondents were women over 50 years of age (mean=54.10; median=54, SD=8.18)

The nutrition of the studied women was determined on the basis of the Body Mass Index. Four categories were adopted : the first included women whose BMI was lower than 18.50 kg/m^2^ - underweight; the second included respondents with the BMI between 18.50 kg/m^2^ and 24.90 kg/m^2^ – normal weight; the third included participants with the BMI between 25.00 kg/m^2^ and 29.90 kg/m^2^ – overweight; and the fourth category included persons whose BMI was equal to or above 30 kg/m^2^ – obesity.

In the study the KomPan Questionnaire was applied which is used to assess the eating habits, frequency of eating in adults, and collect information on respondents’ beliefs on the role of food and nutrition (Jeżewska-Zychowicz et al., 2017). The questionnaire made it possible to make a summarised assessment of diet quality. In order to achieve this two indices were determined: 1) Pro-Healthy Diet Index (pHDI-10) determined on the basis of consumption of 10 groups of food with potentially positive effect on health: 1) wholemeal bread; 2) buckwheat, oatmeal, wholemeal pasta, other grains; 3) milk (flavoured milk, cocoa, coffee); 4) fermented milk drinks; 5) cottage cheese (homogenized, cheese-based desserts); 6) fish; 7) legume-based dishes; 8) fruit; 9) vegetables; 10) white meat-based dishes (poultry, rabbit), 2) Non-Healthy Diet Index-14 – nHDI-14 determined on the basis of consumption of 14 groups of food with potentially adverse effect on health: 1) tinned meat; 2) energy drinks; 3) lard as a bread spread 4) fast foods; 5) wine, alcoholic beverages; 6) sweetened carbonated or still drinks; 7) white rice, white pasta; 8) red meat; 9) fried foods; 10) cheese; 11) butter; 12) sweets; 13) white bread and bakery products; 14) sausages. Then individual frequencies of consumption of certain groups of foods were assigned point value, which made it possible to assess the quality of diet in the scale from 0 to 100 points – the higher the value of the index, the higher the intensity of properties beneficial or adverse for health (1-33 pts. – low; 34-66 pts. – moderate and 67-100 – large).

The questionnaire also made it possible to assess the lifestyle of the studied women and their socioeconomic status. The lifestyle was determined on the basis of the assessment of behaviour related to eating habits, the level of physical activity, smoking and alcohol consumption. The socioeconomic status was described using the following variables: education, professional activity, place of residence, and self-assessment of the financial situation.

In the analysis of the material basic statistical methods were used. For qualitative variables numbers in individual categories were compared, answers were added and fractions of the studied groups were determined. For qualitative variables basic descriptive statistics was used. The relationship between the compared grouping variables: Healthy Diet Index-10 and non-Healthy Diet Index-14 and variables describing socioeconomic status and lifestyle was assessed. For this Spearman’s or Pearson’s rank correlation coefficient was calculated.

In order to check the effect of socioeconomic variables and lifestyle variables on the summary assessment of the diet multiple regression analysis was used. Variables which significantly differentiated the value of the discussed index (p<0.05) or tended to be significant (p<0.10) were qualified for the model.

All calculations were performed using Statistica 10.0 software.

## Results

All studied women lived in a large city (with a population of more than 100 thousand). The majority of them had secondary and university education (38% and 44% of participants, respectively, table 1). More than half of the women had normal body weight, while 39% were overweight, of which 20% were obese. 39% of respondents declared low physical activity at work and 35% in leisure time. On the other hand, the studied women more often had high levels of physical activity in leisure time than at work. Moderate physical activity at work and in leisure time characterised a similar percentage of respondents: 46% and 45%, respectively. More than 85% of participants did not smoke, whereas before the diagnosis this percentage was 55%. Most women described their health as comparable to their peers; in the opinion of 21% of women their health was better and 16% believed that they are in worse health than people of the same age. Women treated for breast cancer mainly assessed their nutritional knowledge as sufficient and good. Only 10% of women believed that their nutritional knowledge is insufficient.

Table 2 presents the frequency of consuming foods with a potentially good effect on health. Most of the studied women declared habitual consumption of wholemeal bread at least once a week (only 1/3 did it at least once a day), milk, fermented milk drinks, cottage cheese and white meat-based dishes. Significantly fewer respondents consumed buckwheat, oatmeal, wholegrain pasta and fish at least once a week – half of the respondents ate these products. Moreover, one in four studied women preferred consuming legume-based dishes at least once a week. Only 2 out of 112 women did not usually eat fruit and vegetables every week; moreover, only one in two respondents did it once a day.

Table 3 shows percentages of women characterised by overconsumption of products or groups of products/dishes classified as foods with adverse health effect. More than half of the studied women consumed the following products or groups of products/foods more than once a week: sausages, white bread, sweets, yellow cheese, fried foods, lard, red meat, white rice and pasta. Overconsumption of fast foods, lard, energy drinks and tinned meat was the least frequent. All studied women consumed at least one of the non-recommended groups of products/dishes more than once a week.

Then individual frequencies of consumption of groups of foods were assigned point values, which made it possible to perform a summary assessment of the quality of diet expressed in the form of the pro-Healthy Diet Index-10 and non-Healthy Diet Index-14. The analysis of the results indicates that the studied women were characterised by a diet with a small intensity of unhealthy properties (nHDI-14 small=100% of respondents) and with a weak influence of protective properties of nutrition (pHDI-10 small=79%, moderate=21% of respondents).

Using a multiple regression analysis a group of biological, social and lifestyle factors influencing the summary assessment of the diet were identified. The following variables were analysed: the level of physical activity in leisure time and at work, smoking (now and in the past), self-assessment of health and nutritional knowledge, education, age, and the BMI. The variables which significantly differentiated the value of the discussed index (p<0.05) or tended to be significant (p<0.10) were qualified for the model. Two models were created: 1) for products/dishes positively affecting health, and 2) for products/dishes adversely affecting health. To this end the summary assessments of the frequency of consumption of individual groups of products/dishes were used: pro-Healthy Diet Index-10 and non-Healthy Diet Index-14. The following variables were introduced to the first model: education, self-assessment of nutritional knowledge and health (n=111). The higher the level of education and the self-assessment of nutritional knowledge and health, the higher the value of pro-Healthy Diet Index. The qualified variables determined 15% of the value of pro-Healthy Diet Index (r=0.38 R^2^=0.15 p<0.001). In the second model the following were placed: self-assessment of nutritional knowledge and health, the BMI, and smoking in the past (n=108). The variables included in the model determined 17% of the value of the non-Healthy Diet Index (r=0.42 R^2^=0.17 p<0.0015). It was demonstrated that the lower the self-assessment of nutritional knowledge and health and the higher the BMI and smoking in the past, the higher the value of non-Healthy Diet Index.

## Discussion

Research indicates that nutritional factors affect development of breast cancer in women, however their relationship depends on both age and the body weight (Dieli-Conwright et al., 2016).

The studied women treated for breast cancer were members of the Poznań Amazon Association. The majority of them were educated, working, aged over 50 years, and the average time since completion of their treatment was more than 5 years.

Age is considered to be one of the main risk factors of breast cancer. The analysis of the collected material indicates that the average age of the studied women was 54 years. This is the age at which most breast cancers are diagnosed in Poland (Didkowska et al., 2017). The scientists have shown that hormonal changes occurring at menopause cause an increase in weight (Munsell et al., 2014). Excessive weight at this time of life disturbs balance in steroid hormones and insulin management. Women with excessive weight are characterised by a significantly higher level of oestrogens compared to women with normal weight. Obesity may cause inflammation which in turn may lead to increased oestrogen production. Oestrogen has been recognised as one of factors contributing to the development of breast cancer (Munsell et al., 2014; Dieli-Conwright et al., 2016).

Being overweight is a complex factor with variable effect on the risk of breast cancer, depending on the age of subjects (Protani et al., 2010, Munsell et al., 2014; Chung et al., 2016). For post-menopausal women obesity is a risk factor, whereas for younger women this factor is virtually insignificant (Dieli-Conwright et al., 2016). Chung et al., (2016) emphasises that overweight women above the age of 60 have a higher risk of the cancer risk. Our results are very alarming. More than 39% of women undergoing breast cancer treatment were overweight which was determined on the basis of body mass index. Protani et al., (2010) in their review of literature demonstrated on the basis of observation studies a higher risk of cancer relapse and death in overweight women and women whose weight increased after the diagnosis compared to women with normal weight. This is also confirmed by the studies of Kroenke et al., (2005), who noted that an increase in weight in women after breast cancer diagnosis was linked to a higher percentage of disease relapse and death in these women. Chan et al., (2014) determined the relative risk of developing breast cancer. On the basis of literature review they analysed the body mass index of more than 200 thousand women with breast cancer. The authors found that an increase in the BMI by 5 kg/m^2^ increases the risk of breast cancer by 17%.

The results of many studies indicate that often women diagnosed with breast cancer try to change their lifestyles in order to improve their health and prevent relapses (Mohammadi et al., 2013; Templeton et al., 2013). In the current study this was manifested in a small percentage of women who smoke (39% gave up smoking after being diagnosed) and drink alcohol. Factors which have positive effect on health include also physical exercise appropriate for age and physical efficiency which is the foundation of the Food pyramid (Kruk and Czerniak, 2013). However, although the awareness in this respect has grown in Poland, low level of physical activity was noted in 35% of the studied women. Specialists indicate that appropriate physical activity adjusted to specific needs and capabilities of an individual prevents overweight and obesity and is conducive to maintaining normal body weight (Kruk and Czerniak, 2013). If one of the factors modifying breast cancer development is overweight and obesity, it seems that physical activity, defined as the regulator of energy balance and an effective way of preventing accumulation of fat reserves, should play a significant part in the lives of all studied women (Czerniak et al., 2014). What is more, the studied women positively assessed their nutritional knowledge. However, the summary assessment of the frequency of consumption of foods positively and adversely affecting health showed that the studied women had a diet with a low intensity of unhealthy properties with a weak effect of protective properties of nutrition. This result shows that the studied women are aware of which products/components of diet negatively affect health, therefore they eliminate them from their menus. On the other hand however, the respondents have insufficient knowledge about products/components of diet which positively affect health.

The results of research show a directly proportional relationship between the consumption of products with a high glycemic index and breast cancer development (Sieri et al., 2007; Gnagnarella et al., 2008; among others). This applies especially women at post-menopausal age (Romieu et al., 2012). Moreover, specialists indicate type 2 diabetes as the disease which is often diagnosed in women treated for breast cancers. Hence the results obtained in the studied group are alarming. One in five studied women consumed sweets at least once a day, and 76% of the respondents had them every week.

**Table1:** Socio-Demographic Characteristics of Researched Women

Categories	Veriables	n	%
education	primary	4	3.57
	basic vocational	16	14.29
	secondary	43	38.39
	high	49	43.75
professional work	working	112	100.00
	does not work	0	0.00
self-assessment of the financial situation	below average	14	12.5
	average	83	74.11
	over average	15	13.39
self-assessment of the level of physical activity	low	39	34.82
	moderate	50	44.64
	hight	23	20.54
self-assessment of physical activity at work	low	43	38.39
	moderate	51	45.54
	hight	18	16.07
smoking	yes	17	15.18
	no	95	84.82
smoking in the past	yes	61	54.46
	no	51	45.54
self-assessment of nutritional knowledge	sufficient	11	9.84
	good	49	43.75
	very good	50	44.64
		2	1.79
BMI index	underweight	3	2.72
	normal	65	58.00
	overweight	22	19.64
	obesity	22	19.64

**Table 2:** Products with Potentially Beneficial Effects on Hhealth - Percentage Characteristics

Question number	Products or product groups	at least once a week	at least once a day
		n/%	n/%
23.	wholemeal bread	84/75,00	34/30,36
25.	buckwheat, oatmeal, whole grain pasta or other coarse grains	60/53,57	14/12,50
31.	milk ( including flavored milk, cocoa, coffee on milk)	76/67,86	48/42,86
32.	fermented milk drinks	90/80,36	27/24,11
33.	curd cheese	88/78,57	7/6,25
37.	melas made by, white meat”	95/84,82	2/1,79
38.	fish	60/53,57	0
40.	dishes from legume seeds	31/27,68	3/2,68
42.	fruits	110/98,21	65/58,04
43.	vegetables	110/98,21	59/52,68

**Table 3:** Products with Potentially Adverse Effects on Health - Percentage Characteristics

Question number	Products or product groups	at least once a week	at least once a day
		n/%	n/%
22.	bakery bread	88/78,57	22/48,21
24.	white rice. plain pasta or small groats	69/61,61	1/0,89
26.	fast food	11/9,82	0
27.	meat or flour fried dishes	77/68,75	3/2,68
28.	butter as addition to bread or dishes. for frying. baking etc.	82/73,21	48/42,86
29.	lard	10/8,93	2/1,79
34.	cheese (including processed cheese. blue cheese)	80/71,43	10/8,93
35.	meats or sausages	89/79,46	13/11,61
36.	dishes made by, red meat”	69/61,61	0
44.	sweets	85/75,89	24/21,43
46.	canned meat	2/1,79	0
51.	sweetened fizzy drinks	21/18,75	4/3,51
52.	energy drinks	3/2,68	0
54.	alcoholic drinks	18/16,07	0

Only less than half of the studied Amazons declared eating fibre-rich products, such as buckwheat, oatmeal, wholegrain pasta in every average week. One in three women declared eating wholemeal bread at least once a day. However, the results of research show a protective action of food fibre in cancers. Women whose diet is rich in food fibre have a lower risk of developing breast cancer compared to respondents whose diets provide little fibre. This is also confirmed by the results of the Nurses’ Health Study and the results of the Diet and Health Study carried out by the National Institutes of Health-AARP (Park et al., 2009).

There is a lot of evidence that biologically active food components, found among others in fruit and vegetables, play an important part in prevention of various diseases, including cancers (Weiner et al., 2010). In the current study more than half of the women undergoing breast cancer treatment did not consume fruit and vegetables at least once a day. And the beneficial role of plant products depends on the consumed amount. It is recommended to provide at least four portions of fruit and vegetables a day (Mattisson et al., 2004). It should be added that two from 112 studied women did not consume fruit and vegetables at least once a day.

The results indicate that in every average week more than 60% of the studied women had red meat and nearly one in two women undergoing breast cancer treatment used butter as bread spread. The results of epidemiological studies confirm increased risk of breast cancer development in women who frequently, e.g. daily, consume red meat and prefer saturated fatty acids in their diets (Taylor et al., 2007; Dieli-Conwright et al., 2016). On the other hand, the protective effect of fatty sea fish in the development of breast cancers has been noted, which in the studied group were consumed by one in two respondents (Janssens et al., 2009). Therefore, also in this respect, the diet of the studied women undergoing breast cancer treatment requires modification.

It seems that there is convincing evidence that the diet of the studied women undergoing breast cancer treatment should be changed, in particular in terms of foods with preventive effect. It is recommended to increase the consumption of food fibre, fruit and vegetables, fatty sea fish providing n-3 polyunsaturated fatty acids and limit the consumption of simple carbohydrates. It is also necessary to introduce physical activity of at least moderate intensity to a daily schedule of every woman participating in the study. A significant effect of nutritional knowledge and health self-assessment of the studied women on both Healthy Diet Index-10 and non-Healthy Diet Index-14 was demonstrated. On the other hand, the awareness resulting from general education is manifested significantly only in case of choice of products positively affecting health. Bad lifestyle habits (excessive intake of energy/being overweight, smoking in the past) contributed to overconsumption of products/dishes with negative health effect.

The results of our analyses show the directions of actions for persons and institutions in charge of disseminating information about the prevention of malignant cancers both in the society and among persons affected by the disease, to ensure that the effects of research are used to lower the risk of breast cancer. On the basis of the results it seems that the studied women had a low level of knowledge about health benefits of appropriate diet and physical activity. Therefore, educating this group of people is important as apart from its therapeutic advantages, it may contribute to an improvement in life quality of the studied women.

## Conclusion

1. The nutrition of the studied women differed from the recommendations for a healthy diet. The studied women had a diet with a low intensity of unhealthy properties and a weak effect of protective nutrition properties.

2. A significant adverse effect of nutritional knowledge and self-assessment of health on pro-Healthy Diet Index-10 and non-Healthy Diet Index-14 was demonstrated.

3. The awareness resulting from general education manifests itself significantly only in case of the choice of products beneficially affecting health. Bad habits related to lifestyle (excessive energy intake/being overweight, smoking in the past) contributed to overconsumption of unhealthy products/dishes.

4. The demonstrated dietary mistakes indicate that actions aiming to promote benefits of undertaking behaviour beneficial for health should also be carried out among women with breast cancer diagnosis after the completion of treatment.

## Funding Statement

Research of the Department of Anthropology and Biometry, University School of Physical Education in Poznań.
